# Progression of Biopsy-Measured Liver Fibrosis in Untreated Patients with Hepatitis C Infection: Non-Markov Multistate Model Analysis

**DOI:** 10.1371/journal.pone.0020104

**Published:** 2011-05-27

**Authors:** Peter Bacchetti, Ross Boylan, Jacquie Astemborski, Hui Shen, Shruti H. Mehta, David L. Thomas, Norah A. Terrault, Alexander Monto

**Affiliations:** 1 Department of Epidemiology and Biostatistics, University of California San Francisco, San Francisco, California, United States of America; 2 Department of Medicine, Johns Hopkins University, Baltimore, Maryland, United States of America; 3 Department of Epidemiology, Johns Hopkins Bloomberg School of Public Health, Baltimore, Maryland, United States of America; 4 Department of Medicine, University of California San Francisco, San Francisco, California, United States of America; 5 Division of Gastroenterology, San Francisco Veterans Affairs Medical Center, San Francisco, California, United States of America; 6 Department of Surgery, University of California San Francisco, San Francisco, California, United States of America; Copenhagen University Hospital, Denmark

## Abstract

**Background:**

Fibrosis stages from liver biopsies reflect liver damage from hepatitis C infection, but analysis is challenging due to their ordered but non-numeric nature, infrequent measurement, misclassification, and unknown infection times.

**Methods:**

We used a non-Markov multistate model, accounting for misclassification, with multiple imputation of unknown infection times, applied to 1062 participants of whom 159 had multiple biopsies. Odds ratios (OR) quantified the estimated effects of covariates on progression risk at any given time.

**Results:**

Models estimated that progression risk decreased the more time participants had already spent in the current stage, African American race was protective (OR 0.75, 95% confidence interval 0.60 to 0.95, p = 0.018), and older current age increased risk (OR 1.33 per decade, 95% confidence interval 1.15 to 1.54, p = 0.0002). When controlled for current age, older age at infection did not appear to increase risk (OR 0.92 per decade, 95% confidence interval 0.47 to 1.79, p = 0.80). There was a suggestion that co-infection with human immunodeficiency virus increased risk of progression in the era of highly active antiretroviral treatment beginning in 1996 (OR 2.1, 95% confidence interval 0.97 to 4.4, p = 0.059). Other examined risk factors may influence progression risk, but evidence for or against this was weak due to wide confidence intervals. The main results were essentially unchanged using different assumed misclassification rates or imputation of age of infection.

**Discussion:**

The analysis avoided problems inherent in simpler methods, supported the previously suspected protective effect of African American race, and suggested that current age rather than age of infection increases risk. Decreasing risk of progression with longer time already spent in a stage was also previously found for post-transplant progression. This could reflect varying disease activity, with recent progression indicating active disease and high risk, while longer time already spent in a stage indicates quiescent disease and low risk.

## Introduction

Chronic infection with hepatitis C virus (HCV) has been estimated to affect 3.2 million persons in the United States and 130 million worldwide and is a leading cause of liver failure and the need for liver transplant [1], [2]. One way of assessing liver damage known as fibrosis is to categorize liver biopsies into fibrosis stages using established scales that range from no damage (stage 0) to cirrhosis [3]. Although such fibrosis staging is widely used clinically, statistical analysis of biopsy-measured fibrosis progression poses considerable challenges. First, the stages are ordered but are not numeric, meaning that differences between consecutive stages are not necessarily equivalent in any meaningful sense. Second, biopsies are too invasive and expensive to perform frequently. Many patients in research studies provide only one observed stage. When multiple observations are available, they are usually widely spaced (e.g., 5 years apart), and an observed progression could have occurred at any time between biopsies, which leaves the exact time of progression unknown. Third, observed fibrosis stage is often misclassified, both because reading of biopsy specimens is not perfectly standardized and because biopsies may not accurately represent the overall state of the entire liver [4]. Finally, most patients available for study have been infected with HCV at some unknown time in the past, and the usual practice of imputing this time based on reported histories of risk factors can be inaccurate [5].

Methods for multistate modeling [6], [7], such as implemented in the “msm” package for R (available at http://cran.r-project.org/web/packages/msm/index.html), deal with many of these difficulties and have been used to analyze fibrosis stage data [8], [9], but they make the strong simplifying assumption that previous history of progression does not impact current risk of progression—the so-called memoryless or Markov assumption. For HCV, however, there is considerable interest in whether slow progression up to the present predicts low risk of progression in the future. A new method for multistate modeling without Markov assumptions was recently applied to fibrosis progression following liver transplant (where time of infection of the new liver is known). Here, we apply that method [10] to data from chronically infected patients from three studies, using multiple imputation [11] to account for uncertainty about time of HCV infection.

## Methods

### Ethics Statement

We report here a secondary analysis of fully de-identified data, including no dates more specific than calendar year. This was approved by the University of California at San Francisco Committee on Human Research. The original source studies (see below) obtained written informed consent from participants to have their data stored and analyzed for research purposes, and they were approved by the University of California at San Francisco Committee on Human Research and the Johns Hopkins Bloomberg School of Public Health Review Board.

### Objectives

We sought to assess the impact of potential risk factors on fibrosis progression, while avoiding questionable assumptions and accounting for fibrosis misclassification and uncertainty about duration of HCV infection. Particular interest focused on how history of progression up to a given time predicts current risk of progression. Human immunodeficiency virus (HIV) infection [12], African American race [9], and age [5] were predictor variables of particular interest.

### Data Sources

We report here new analyses of previously-collected data from three studies: the AIDS Link to Intravenous Experience (ALIVE) study [13], [14], [15]; the Hepatitis C and Alcohol Study (HALS) [16]; and the San Francisco Veterans Affairs Medical Center (SFVA) liver studies cohort [17]. For this analysis, we excluded participants with chronic hepatitis B infection or hepatocellular carcinoma, and we excluded biopsy results that were after interferon treatment or liver transplant. The ALIVE study had fibrosis staged on both the Metavir [18] and Ishak [19] scales; a cross-tabulation of stages showed near-perfect correspondence of Ishak 0 with Metavir 0, Ishak 1 or 2 with Metavir 1, Ishak 3 with Metavir 2, Ishak 4 or 5 with Metavir 3, and Ishak 6 with Metavir 4 (cirrhosis). We used this correspondence to convert Ishak scores in the HALS study to Metavir scores. SFVA participants had biopsies staged from 0–4 using the Batts-Ludwig scale [20]. We treated this as equivalent to Metavir stages for analysis purposes because both are 0 to 4 ratings with similar criteria for each stage and because a study directly comparing the methods on the same biopsies found exact agreement in 49 of 50 cases [21]. All biopsies were obtained prospectively after study enrollment. (A previous analysis of SFVA data [17] excluded pre-1997 biopsies because they lacked the needed type of data on alcohol use.)

### Statistical methods

#### Model

To preserve the advantages of multistate modeling while avoiding questionable Markov assumptions and allowing use of covariates that change over time, we used a new method implemented in the R package *mspath*, which is available at http://cran.r-project.org/web/packages/mspath/index.html. Technical details of this method have been described elsewhere [10]. The method assumes the following outline of how disease progresses:

1. Each person starts at stage 0 at the time of HCV infection.
2. Time after infection is divided into discrete time steps (such as age in years).
3. At each time step, the person either remains at the same stage or progresses to the next higher stage.
4. The risk of progression at each time step is determined by the progression history up to that point, along with covariates, including current values of covariates that may have changed over time (termed *time-varying covariates*).

The method considers for each person every specific history of progression over time (or *path*) that could have produced the observed fibrosis stage(s). For example, a person with an observed stage of 2 at time step 5 could have 1) progressed to stage 1 at step 1, to stage 2 at step 3, stayed in stage 2 until step 5 and then been accurately observed, or 2) progressed to stage 1 at step 2, to stage 2 at step 5 and then been accurately observed, or 3) progressed to stage 1 at step 5 and then been misclassified as stage 2, and so on (too many possibilities to list, even in this simple case). Models that include effects of progression history up to a given point, and effects of time-varying covariates, can be applied to each specific path, and the likelihood of having observed the actual data is then calculated by summing over all the possible specific paths. Estimated covariate effects are obtained as those that maximize the likelihood of the observed data, a standard statistical approach to estimation. The influence of covariates on the probability of progression to the next stage is modeled on the log-odds scale, so we present estimated effects as odds ratios. We defined the time scale as current age in years minus age in years at time of HCV infection, and most models used time steps of 1.5 years (to keep computational burden manageable). In a sensitivity analysis, we re-estimated one model using 1-year time steps. We also excluded biopsies occurring 40 or more years after HCV infection (again to keep computational burden manageable).

#### Misclassification of stage

To account for the reality that observed stage at a given time may differ from the person's true stage at that time, we included misclassification probabilities in the models. For most models, we assumed the optimistic misclassification probabilities shown in Table 1. These are from an analysis of several studies specifically focused on misclassification of fibrosis stage from liver biopsies [4]. In a sensitivity analysis, we also re-estimated one model using the more pessimistic misclassification probabilities in Table 1, which are also from the earlier analysis [4].

**Table 1:** Misclassification probabilities assumed for analyses.

		Probability of Observed Stage Given True Stage									
		Optimistic*					Pessimistic†				
		0	1	2	3	4	0	1	2	3	4
True Stage	0	0.81	0.19	0	0	0	0.74	0.26	0	0	0
	1	0.07	0.73	0.19	0	0	0.24	0.54	0.22	0	0
	2	0	0.10	0.80	0.09	0	0	0.19	0.65	0.16	0
	3	0	0.03	0.23	0.67	0.07	0	0.10	0.45	0.37	0.08
	4	0	0	0	0.08	0.92	0	0.01	0.07	0.25	0.67

*From Table 1, line 4 of reference [4].

† From Table 3 of reference [4].

#### Predictors based on past progression history

We investigated possible departures from the usual Markov assumptions by assessing four predictor variables that the mspath program defines for each step of each path, reflecting progression history up to that point:

Time in stage—the amount of time already spent in the current stage. A negative coefficient or odds ratio <1 for this variable indicates that risk of progression is less when a longer time has already been spent in the stage without progressing; this might occur if having recently progressed to the current stage tends to indicate that disease is active, creating higher risk of continuing progression, while having been in the stage a long time tends to indicate quiescent disease and lower risk. A positive coefficient or odds ratio >1 indicates that risk of progression is higher when a longer time has already been spent in the current stage; this might occur if underlying disease is steady and incremental so that it eventually accumulates enough to manifest as progression to the next stage.

Log_e_(*c*+time in stage), where *c* is a specified positive number that prevents taking the logarithm of zero the first time a path is in a new stage (we used *c* = half the step size in all analyses). This allows a different shape for the influence of time in stage on progression risk. Its qualitative interpretation is the same as noted above for time in stage.

Total time in all previous stages—the amount of time that it took to reach the current stage. A negative coefficient or odds ratio <1 for this variable indicates that risk of progression is less if the person has previously been progressing more slowly (took longer to reach the current stage). A positive coefficient or odds ratio >1 would indicate that those previously progressing more slowly are now at higher risk. (This variable is not used in modeling progression from stage 0 to 1, because there are no previous stages.)

Log_e_(*c*+total time in previous stages), where *c* is the specified positive constant described above. This allows a different shape for the influence of time in previous stages on progression risk.

#### Other predictors

We evaluated a number of other factors that may influence fibrosis progression. The three studies selected participants in different ways from different populations, so we controlled for study in all models by including indicator variables for ALIVE and for HALS. This was important for preventing spurious apparent associations due to confounding with source study. We allowed the effects of study to be *stage-varying*, i.e., to differ for progression between different stages, because the simplifying assumption that the effect was identical for all 4 transitions between stages did not fit the data nearly as well. Other predictors were initially evaluated as having the same effect on all transitions; they are listed below:

Sex—male compared to female.

Race/ethnicity—classified as Caucasian, African America, Hispanic, and other. Because of previous findings concerning African Americans, we also evaluated this as African American compared to all other categories lumped together.

HIV—we determined coinfection with HIV at each time step, based on age at HIV infection imputed as described in the next section. Because treatment for HIV changed dramatically over time [22], we also examined whether the effect of HIV differed in different calendar periods: before 1996 versus 1996 and later; and before 1996 versus1996–2000 versus after 2000.

Primary reported HCV infection risk factor—classified as injection drug use for participants reporting any injection drug use; otherwise, we classified it as receipt of blood transfusion or as needlestick if those were reported. All others were lumped together as “Other/none”.

Tobacco smoking—collected only during study participation. We classified this as yes or no based on any reported smoking, and assumed that the earliest report also applied back to age 16.

Alcohol consumption—the HALS study collected a comprehensive alcohol consumption history, but the other studies only provided information on recent consumption collected during study participation. For each year of age of HALS participants, we categorized alcohol consumption as “None” if the age was in a period of reported alcohol abstinence, as “Moderate” if they reported drinking less than 3 drinks per day on less than 20 days per month or less than 5 drinks per day on less than 4 day per month, or as “Heavy” otherwise. For the other two studies, we approximated similar definitions using available data and assumed that the earliest measures applied back to age 21. Because this is likely to be inaccurate, primary analyses of alcohol only used HALS participants.

Injection drug use—based on reported ages of first and last injection drug use, we determined whether each participant was using injection drugs at each time step.

Body mass index—this was defined as weight in kilograms divided by the square of height in meters, which were only collected during study participation. We assumed that the earliest value also applied back to the age of HCV infection.

Current age—evaluated at each time step as a time-varying covariate.

Age at HCV infection—evaluated as a fixed covariate, multiply imputed as described in the next section.

#### Multiple imputation

We applied a strategy known as multiple imputation [11], because exact values were generally unavailable for age at HCV infection and age at HIV infection, and some observations had missing data for alcohol consumption, smoking, and body mass index. This approach is more valid than assuming that infection occurred at the reported age of first risk factor (which has typically been used for age at HCV infection [5]) or the common practice of simply deleting observations that have a missing value for any covariate. For risk factor modeling, we generated 5 data sets, each replacing missing covariates with values randomly imputed from models built using the non-missing data, along with imputed ages of HCV and HIV infection from external analyses (see next paragraph). We then analyzed each and combined the results of the 5 analyses using established methods to obtain overall estimates and standard errors [11]. In some cases, estimated log odds ratios in some or all imputed data sets were effectively infinite, causing methods based on standard errors in multiple imputation to break down. We therefore note likelihood ratio p-values and profile likelihood confidence bounds for some results. We use the term *deviance* to denote twice the negative log likelihood, which is the quantity used in likelihood ratio tests; a difference in deviance of 3.84 has p = 0.05 for comparing a base model to one with one additional parameter.

We imputed 5 values of age at HCV infection for each participant by putting their risk factor histories and age first known to be infected into an external model of risk. The model has been reported previously [5] and was based on reported injection drug use history and other characteristics; it was built using data from 4623 street-recruited injection drug users. As a sensitivity analysis, we repeated some models using 5 imputed data sets based on a model of HCV infection risk built by the same methods but using data from 2248 mostly HIV-infected women [5]. The reference [5] fully describes both models, includes figures illustrating the effects of age and calendar time, and gives the exact code that we used to obtain the fitted probabilities of infection at each age for each person in a supplemental file at http://www.biomedcentral.com/content/supplementary/1471-2334-7-145-s2.pdf. For purposes of summarizing the available data, we also imputed one additional value as the conditional mean of the probability distribution of age at HCV infection given each participant's first age known infected and risk factors. We imputed age of HIV infection using age first known to be infected and the estimated distribution of numbers of infections among injection drug users over calendar time from a national study [23], assuming no risk before age 13 or before the year 1980. We then imputed other missing predictors using the Markov-chain-Monte-Carlo method in the Statistical Analysis System's (SAS Institute, Cary, NC, version 9.1.3) MI procedure, separately for each study, with all available variables included in the process. To approximate the recommended practice of including the outcome as one of the variables used to impute missing predictors [24], we also included a variable defined as the first observed fibrosis score divided by years since imputed HCV infection.

#### Predictor selection

Because simultaneous inclusion of all potential risk factors in a single model would not be computationally feasible or statistically reliable, we sought to build a parsimonious multivariate model that included risk factors that had the strongest evidence for an influence on progression. We then examined the effects of the remaining potential risk factors when added to this model. Because of the high computational burden of our method, we performed some preliminary exploration of models using the additional single imputation based on conditional mean age of HCV infection, but we found that this differed too much from multiple imputation analyses using the 5 randomly imputed data sets. We therefore used full multiple imputation for confirmation of predictor selection. We included source study because it was a potential confounder of other effects, and we included log(0.75+years in stage) because it was of primary interest and appeared to strongly influence progression risk. When added to a model including stage-varying study effects and log(0.75+years in stage), African American race (versus all others) appeared to be an important predictor, based on the p-value and direction and magnitude of the estimated effect. We therefore report this primary model in detail, along with the estimated effects that other predictors had when added to this model. We also evaluated substituting each of the other 3 predictors based on progression history for log(0.75+years in stage), and adding the time-in-previous-stages predictors to the primary model.

#### Special handling of age variables

The estimated effects of the two age variables listed above, current age at each time step and age at HCV infection, may be subject to bias [5]. When the imputed age of HCV infection is too early, this will make progression look slower than it really was, inducing a spurious protective effect of younger age at infection. When imputed age of infection is too late, this will make progression look faster than it really was, making older age at infection appear to increase risk, which is the same spurious effect. Thus, any error in either direction creates the same bias, and multiple imputation may not do much to mitigate this problem. Little change in the estimated effect was observed with multiple imputation in a previous study [9]. For current age at each time step, the impact may be more subtle because this variable is known rather than imputed with some error. Bias may nevertheless occur because age at infection determines which ages are assumed to be part of followup during which progression could have occurred. A too-early imputation of age at HCV infection will make progression look slower and cause spurious inclusion of some younger current ages in the post-infection followup time, while a too-late imputation will make progression look faster and will cause only older current ages to be included as post-infection. We therefore did not include current age or age at HCV infection in the primary models evaluated as described in the previous paragraph. We added each, and both, to the primary model, and we also performed some simulations to evaluate the potential bias. We used the primary fitted model without age effects to generate simulated observations of fibrosis stage, at the same times as the original observations, using an additional independent set of imputed ages at HCV infection. We did this independently for each of the 5 original imputed data sets, generating 5 new simulated data sets with realistic amounts of error in the imputed HCV infection ages and with no actual association of current age or age at HCV infection with rate of progression. We then fit the primary model plus current age and the primary model plus age at HCV infection to the simulated data; any apparent effects in these models are due to bias and therefore provide some indication of how much bias may be present. We then repeated the entire process using another independently imputed set of assumed actual HCV infection ages. Performing a large number of such simulations, however, would not be computationally practical.

## Results

### Study participants

There were 1082 participants available for study, with 1284 biopsies. For 20 of them, their first biopsy was ≥40 years after their mean predicted age of HCV infection. Because our limitation of followup time to 39 years is likely to exclude these from most imputed data sets, Tables 2 and 3 summarize characteristics of the remaining 1062. The randomly imputed ages of HCV infection are more variable than the means and therefore have more that were long ago, so the 5 imputed data sets ranged from 1015 to 1027 participants included.

**Table 2:** Summary of time-related characteristics, by source study.

	ALIVE N (% of 236)	HALS N (% of 202)	SFVA N (% of 624)	Total N (% of 1062)
Age at HCV Infection* <15	6 (2.5)	8 (4.0)	10 (1.6)	24 (2.3)
15–19	92 (39.0)	71 (35.2)	145 (23.2)	308 (29.0)
20–24	103 (43.6)	85 (42.1)	249 (39.9)	437 (41.2)
25–29	24 (10.2)	32 (15.8)	162 (26.0)	218 (20.5)
≥30	11 (4.7)	6 (3.0)	58 (9.3)	75 (7.1)
Age at last biopsy <30	2 (0.9)	6 (3.0)	2 (0.3)	10 (0.9)
30–39	33 (14.0)	33 (16.3)	36 (5.8)	102 (9.6)
40–49	141 (59.8)	102 (50.5)	198 (31.7)	441 (41.5)
50–59	57 (24.2)	58 (28.7)	335 (53.7)	450 (42.4)
≥60	3 (1.3)	3 (1.5)	53 (8.5)	59 (5.6)
Year of HCV infection* Pre-1966	0 (0.0)	3 (1.5)	12 (1.9)	15 (1.4)
1966–1970	42 (17.8)	28 (13.9)	156 (25.0)	226 (21.3)
1971–1975	66 (28.0)	50 (24.8)	230 (36.9)	346 (32.6)
1976–1980	65 (27.5)	60 (29.7)	162 (26.0)	287 (27.0)
1981–1990	59 (25.0)	56 (27.7)	60 (9.6)	175 (16.5)
After 1990	4 (1.7)	5 (2.5)	4 (0.6)	13 (1.2)
Year of last biopsy 1992–1995	0	00.00	11 (1.8)	11 (1.0)
1996–1999	83 (35.2)	2 (1.0)	134 (21.5)	219 (20.6)
2000–2003	82 (34.7)	189 (93.6)	290 (46.5)	561 (52.8)
2004–2008	71 (30.1)	11 (5.4)	189 (30.3)	271 (25.5)
Years, infection to last biopsy* <20	49 (20.8)	39 (19.3)	38 (6.1)	126 (11.9)
20–24	60 (25.4)	56 (27.7)	125 (20.0)	241 (22.7)
25–29	69 (29.2)	58 (28.7)	212 (34.0)	339 (31.9)
30–34	43 (18.2)	40 (19.8)	173 (27.7)	256 (24.1)
35–39	15 (6.4)	9 (4.5)	76 (12.2)	100 (9.4)

*Based on single imputation of age at HCV infection (see text).

Abbreviations: ALIVE: AIDS Link to Intravenous Experience study [13], [14], [15]; HALS: Hepatitis C and Alcohol Study [16]; SFVA: San Francisco Veterans Affairs Medical Center liver studies cohort [17]; HCV: hepatitis C virus.

**Table 3:** Summary of other characteristics, by source study.

	ALIVE N (% of 236)	HALS N (% of 202)	SF VA N (% of 624)	Total N (% of 1062)
Sex Female	73 (30.9)	62 (30.7)	63 (10.1)	198 (18.6)
Male	163 (69.1)	140 (69.3)	561 (89.9)	864 (81.4)
Race/Ethnicity Missing	0 (0.0)	2 (1.0)	0 (0.0)	2 (0.2)
White	15 (6.4)	84 (41.6)	419 (67.2)	518 (48.8)
Black	215 (91.1)	60 (29.7)	134 (21.5)	409 (38.5)
Hispanic	5 (2.1)	37 (18.3)	38 (6.1)	80 (7.5)
Other	1 (0.4)	19 (9.4)	33 (5.3)	53 (5.0)
HIV-infected No	155 (65.7)	160 (79.2)	560 (89.7)	875 (82.4)
Yes	81 (34.3)	42 (20.8)	64 (10.3)	187 (17.6)
HCV risk factor Injection drug use	236 (100)	135 (66.8)	345 (55.3)	716 (67.4)
Transfusion	0 (0)	25 (12.4)	58 (9.3)	83 (7.8)
Needlestick	0 (0)	5 (2.5)	36 (5.8)	41 (3.9)
Other/none	0 (0)	37 (18.3)	185 (29.7)	222 (20.9)
Smoking Missing	0 (0.0)	0 (0.0)	190 (30.5)	190 (17.9)
No	20 (8.5)	73 (36.1)	54 (8.7)	147 (13.8)
Yes	216 (91.5)	129 (63.9)	380 (60.9)	725 (68.3)
Alcohol use* Missing	0 (0.0)	0 (0.0)	120 (19.2)	120 (11.3)
None	34 (14.4)	4 (2.0)	107 (17.2)	145 (13.7)
Moderate	47 (19.9)	20 (9.9)	36 (5.8)	103 (9.7)
Heavy	155 (65.7)	178 (88.1)	361 (57.9)	694 (65.4)
Body mass index (kg/m^2^)* Missing	95 (40.3)	15 (7.4)	211 (33.8)	321 (30.2)
<25	88 (37.3)	68 (33.7)	135 (21.6)	291 (27.4)
25–30	22 (9.3)	78 (38.6)	156 (25.0)	256 (24.1)
30 & up	31 (13.1)	41 (20.3)	122 (19.6)	194 (18.3)
Number of biopsies analyzed 1	130 (55.1)	202 (100)	571 (91.5)	903 (85.0)
per participant 2	65 (27.5)	0 (0)	53 (8.5)	118 (11.1)
3	41 (17.4)	0 (0)	0 (0.0)	41 (3.9)
Highest fibrosis stage observed 0	67 (28.4)	55 (27.2)	214 (34.3)	336 (31.6)
1	126 (53.4)	112 (55.5)	170 (27.2)	408 (38.4)
2	23 (9.8)	19 (9.4)	139 (22.3)	181 (17.0)
3	8 (3.4)	16 (7.9)	63 (10.1)	87 (8.2)
4	12 (5.1)	0 (0.0)	38 (6.1)	50 (4.7)

*Highest value in available data since imputed age of HCV infection.

Abbreviations: ALIVE: AIDS Link to Intravenous Experience study [13], [14], [15]; HALS: Hepatitis C and Alcohol Study [16]; SFVA: San Francisco Veterans Affairs Medical Center liver studies cohort [17]; HIV: human immunodeficiency virus; HCV: hepatitis C virus.

### Progression model and risk factors

**Figure 1:**
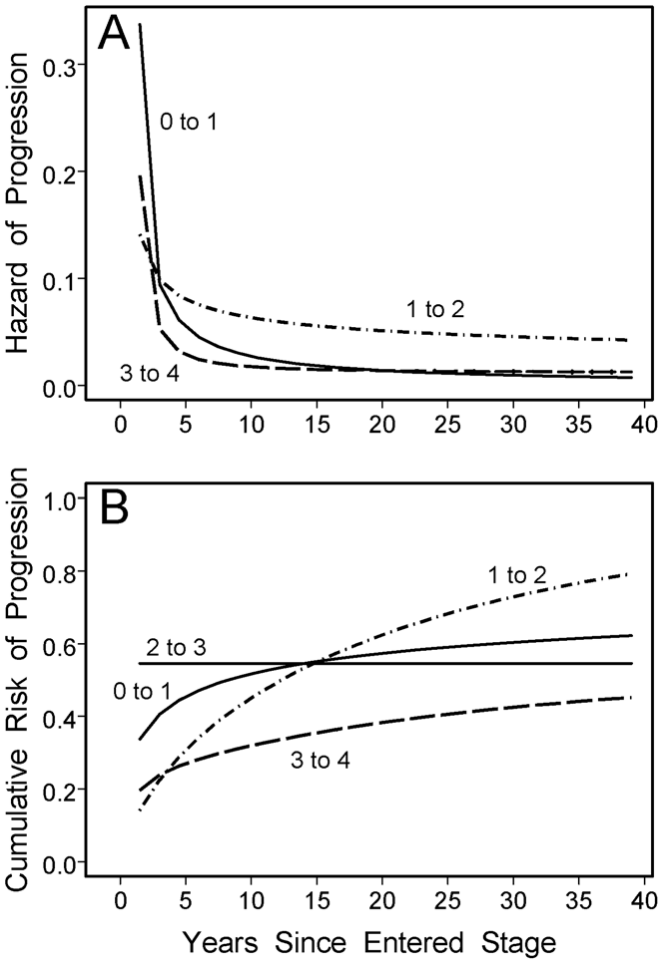
Baseline progression risk for the model in the top part of **Table 4**, for a non-black participant in the San Francisco Veterans Affairs Medical Center liver studies cohort [17]. (A) Risk of progression at a time step of 1.5 years given no progression at earlier time steps (*hazard* of progression). All transitions have decreasing hazard, reflecting the odds ratios <1 in Table 4 for years in stage. For the transition from stage 2 to 3, the estimated hazard of progression is 0.55 for the first step and 0 at all later times; this is not shown to avoid compression of the vertical scale for the other transitions. (b) Cumulative risk of progression. The cumulative risk in the first time step is equal to the hazard; at later time steps, it is equal to the previous cumulative risk plus the current hazard times (1 – previous cumulative risk). The cumulative risk therefore increases by less than the current hazard when the previous cumulative risk is already substantial.

Our primary fitted progression model is described by Figure 1 and the top part of Table 4. The odds ratios for years in stage are somewhat complicated to interpret. For example, for the 0 to 1 transition, the odds ratio of 0.39 implies that the estimated odds of progressing drop by a factor of 0.39 if log_e_(0.75+years in stage) increases by 1 unit, which means that (0.75+years in stage) increases by a factor of *e*≈2.72. This would be the case if years in current stage increase from 1.5 to 5.4, for example. We use Figure 1 to more simply illustrate the estimated baseline progression risk by time step based on fitted intercept terms and the odds ratios for log_e_(0.75+years in stage). We show the pointwise averages of the fitted curves for the five imputed data sets, because curves defined by average parameter values would be distorted by instances of effectively infinite estimated parameters for some of the imputed data sets. All the estimates had risk of progression decreasing as time already spent in the stage increased. From Table 4, we see that evidence for this phenomenon was statistically significant for the stage 0 to 1 and 2 to 3 transitions. The decrease had a large p-value for the 1 to 2 transition. The overall strength of evidence is unclear for the 3 to 4 transition, because two of the imputed data sets produced effectively infinite estimates (risk drops to zero after progression has been avoided for one step) with likelihood ratio p-values of 0.021 and 0.028. The protective effect of African American race versus all other groups was in the expected direction with a small p-value. Allowing African American race to have different effects for the different transitions did not produce a statistically significant improvement in any of the 5 imputed data sets (all p≥0.70); the estimates for all transitions were protective and similar to the overall estimate except for stage 3 to 4 (odds ratio 1.35, 95% confidence interval 0.35 to 5.2, p = 0.66).

**Table 4:** Primary progression model, and estimated effects of total time in previous stages when added to the primary model.

		Odds	95% confidence interval		
Predictor	Transition	Ratio^a^	Lower	Upper	P-value
Log_e_(0.75+years in stage)	0 to 1^b^	0.39	0.23	0.65	0.0004
	1 to 2	0.72	0.37	1.42	0.35
	2 to 3^c^	0	0	0.84	0.028
	3 to 4^d^	0.52	0.06	4.6	0.56
ALIVE study (vs SFVA study)	0 to 1	1.38	0.97	1.97	0.073
	1 to 2	0.21	0.11	0.39	<0.0001
	2 to 3^e^	20.0	0.07	+∞	0.30
	3 to 4	4.3	0.92	20.4	0.063
HALS study (vs SFVA study)	0 to 1	1.66	1.12	2.4	0.011
	1 to 2	0.16	0.09	0.30	<0.0001
	2 to 3^f^	+∞			
	3 to 4^f^	0			
African American (vs all others)	All	0.75	0.60	0.95	0.018
		Effects when added to above primary model			
Years in all previous stages (per 1 year)	1 to 2^g^	0.16	0.02	1.05	0.056
(per 5 years)	2 to 3^h^	2.1	0.31	14.3	0.45
(per 5 years)	3 to 4	1.26	0.05	29.7	0.89

a Odds ratios indicate the estimated effects of the predictors on the risk of progression to the next stage at any given time step. Odds ratios below 1.0 indicate lowered risk; ratios above 1.0 indicate increased risk.Based on 4 imputed data sets; the fifth had estimated odds ratio (OR) = 0 with likelihood ratio (LR) p-value <0.0001.

b Based on 4 imputed data sets; the fifth had estimated odds ratio (OR) = 0 with LR p-value <0.0001.

c All imputed data sets had estimated OR = 0; the largest of the 5 LR p-values and the corresponding profile likelihood confidence bound are shown.

d Based on 3 imputed data sets; the other two estimated odds ratios were 0.13 and 0.15 with LR p-values of 0.028 and 0.021, but these were close to degenerate, with much larger estimated standard errors and deviance nearly identical at OR = 0.

e Although the estimated OR was finite, the estimates in all 5 imputed data sets appeared to be nearly degenerate, with large standard errors and deviance at OR = +∞ nearly as good as at the finite estimated values.

f Estimates in all 5 imputed data sets were degenerate. As these are not parameters of interest, we did not pursue LR p-values or profile likelihood confidence bounds.

g Based on 4 imputed data sets; the fifth had estimated OR = 0 with likelihood ratio p-value <0.0001.

h Based on 3 imputed data sets; the other two had estimated OR = +∞ with LR p-values 0.021 and 0.082.

Abbreviations: ALIVE: AIDS Link to Intravenous Experience study [13], [14], [15]; HALS: Hepatitis C and Alcohol Study [16]; SFVA: San Francisco Veterans Affairs Medical Center liver studies cohort [17].

Substituting the untransformed years in stage for log_e_(0.75+years in stage) produced similar models but with slightly worse fits overall (deviance greater by 1.7 on average over the 5 data sets). Substituting either variable based on total time in previous stages produced worse fits (deviance worse by at least 17 for every data set). When added to the primary model, longer time in stage 0 appeared to greatly reduce progression risk for the stage 1 to 2 transition, indicating the plausible phenomenon of slower progression through stage 0 predicting slower progression through stage 1. The evidence for this was somewhat stronger than shown in Table 4, because one of the imputed data sets had odds ratio = 0 (a degenerate estimate) with a likelihood ratio p-value <0.0001. For the 2 to 3 and 3 to 4 transitions, the effect of total time spent in previous stages was estimated to be in the opposite direction, but with wide confidence intervals. For the 2 to 3 transition, the evidence for increased risk with longer time spent previous stages is also stronger than indicated in Table 4 because two of the five data sets produced effectively infinite odds ratios, with likelihood ratio p-values of 0.021 and 0.082.

Table 5 shows estimated effects of other potential predictors when controlled for all the terms in the primary model. The effect of African American race was slightly weaker versus Caucasians than versus all others (Table 4). Coinfection with HIV appeared only slightly risky overall, with a wide confidence interval that included substantial increased risk. Allowing the effect of HIV to change with the advent of widely available highly active anti-retroviral therapy in 1996 [22] produced a much higher estimated risk in this era, but this did not quite reach p<0.05; the estimated effect of HIV before 1996 became very uncertain. Further subdividing HIV effects by calendar time resulted in highly uncertain estimates. The estimated effect of heavy alcohol consumption was in the expected direction, but modest and not statistically significant; the upper confidence bound, however, allows for a fairly substantial increased risk.

**Table 5:** Estimated effects of each other predictor when added singly to the primary model from Table 4.

		Odds	95% confidence interval		
Predictor	Value	Ratio	Lower	Upper	P-value
Sex	Male	1.21	0.94	1.57	0.14
Race/ethnicity^a^	African American	0.79	0.62	1.01	0.055
(vs Caucasian)	Hispanic	1.26	0.91	1.75	0.17
	Other	1.23	0.85	1.79	0.27
HIV-infected^b^	Yes	1.17	0.64	2.1	0.61
HIV-infected, by treatment era^b^	to 1995	0.68	0.17	2.7	0.58
(vs uninfected)	1996 on	2.1	0.97	4.4	0.059
HIV-infected, by treatment era^b^	to 1995	0.37	0.00	568	0.79
(vs uninfected)	1996–1999	3.0	0.57	16.1	0.19
	2000 on	1.49	0.34	6.6	0.60
Reported HCV risk factor	Transfusion	0.99	0.72	1.37	0.96
(vs injection drug use)	Needlestick	0.88	0.61	1.28	0.50
	Other/None	1.21	0.96	1.53	0.11
Smoking	Yes	1.07	0.83	1.40	0.59
Alcohol consumptionb,c	Moderate	0.99	0.52	1.88	0.99
(vs none)	Heavy	1.16	0.72	1.89	0.54
Injection drug use^b^	Yes	0.92	0.69	1.23	0.58
Body Mass Index	per 5 Kg/m^2^	1.05	0.97	1.14	0.24
Body Mass Index	25–30	1.09	0.88	1.36	0.42
(vs <25)	>30	1.19	0.95	1.50	0.14

a This is an alternative finer breakdown instead of an addition to the primary model.

b These are time-varying covariates, with potentially differing values at each time step.

c This model was fitted on HALS participants only, because other studies lacked complete histories.

Abbreviations: HIV: human immunodeficiency virus; HCV: hepatitis C virus; HALS: Hepatitis C and Alcohol Study [16].

### Sensitivity analyses

Table 6 shows the main results of interest produced by repeating the primary model from Table 4 with three alterations, as indicated in the body of the table. The key results, decreasing risk with longer time already spent in a stage and the protective effect of African American race, remain very similar. For the case where age of HCV infection was imputed using a different model, we also evaluated the estimated effects of HIV when added to the primary model. The estimated effect of HIV at any time remained similar (odds ratio 1.24, 95% confidence interval 0.84 to 1.83, p = 0.27), as did the estimated effect of HIV in the year 1996 and later (odds ratio 1.98, 95% confidence interval 0.98 to 4.0, p = 0.058). Changing the time step to 1 year or using the more pessimistic misclassification probabilities increased the computational burden by 3- to 5-fold, to over a full day of processing time per imputed data set in some cases. This made more extensive sensitivity analyses and pursuit of likelihood ratio p-values and profile likelihood confidence intervals too difficult to be worthwhile, particularly given the reassuring initial findings.

**Table 6:** Results of sensitivity analyses for the primary model from Table 4 on predictors of interest.

		Odds	95% confidence interval		
Predictor	Transition	Ratio	Lower	Upper	P-value
One-year time steps instead of 1.5 year-time steps					
Log_e_(0.75+years in stage)	0 to 1^a^	0.40	0.25	0.66	0.0003
	1 to 2	0.77	0.40	1.47	0.42
	2 to 3^b^	0			
	3 to 4^c^	0.51	0.09	2.90	0.44
African American (vs all others)	All	0.76	0.60	0.96	0.019
Pessimistic misclassification probabilities instead of optimistic, from Table 1					
Log_e_(0.75+years in stage)	0 to 1^a^	0.26	0.06	1.20	0.084
	1 to 2	0.77	0.37	1.59	0.48
	2 to 3^b^	0			
	3 to 4^d^	0			
African American (vs all others)	All	0.71	0.53	0.95	0.020
Age of HCV infection imputed from alternative model					
Log_e_(0.75+years in stage)	0 to 1^c^	0.37	0.22	0.63	0.0003
	1 to 2	0.70	0.40	1.21	0.20
	2 to 3^b^	0			
	3 to 4^e^	0.46	0.06	3.46	0.45
African American (vs all others)	All	0.77	0.63	0.94	0.011

a Based on 4 imputed data sets; the fifth had estimated odds ratio (OR) = 0.

b All imputed data sets had estimated OR = 0.

c Based on all 5 imputed data sets; none had estimated OR = 0.

d Three of the imputed data sets had estimated OR = 0, precluding meaningful combination of just the remaining 2.

e Based on 4 imputed data sets; the fifth had a nearly degenerate estimated OR = 0.09 with an effectively infinite standard error, precluding synthesis with the others.

Abbreviations: HCV: hepatitis C virus.

### Evaluation of age effects

Table 7 shows the results of several analyses of age effects. Both older current age at each time step and older age at HCV infection showed strong evidence of increasing progression risk. When both were included in the same model, however, current age appeared to be the important factor. There was some collinearity between the two, particularly for participants with shorter followup after HCV infection, so the uncertainty in both estimated effects is large in the model that includes both. This indicates that, in our data, neither improves the fit to the data very much when added to the model that already includes the other. Testing the linearity assumption for current age by adding a quadratic term produced a p-value of 0.27, indicating no strong evidence for non-linearity; the estimated curvature was negative, indicating a slowing in the rate of increased risk as age increases. Allowing the effect of current age to differ for the different transitions between stages did not appear to produce substantially improved fits to the data. The average improvement in the deviance was 4.6, which would not be unusual by chance alone with the addition of 3 parameters (4 age effects instead of one); one imputed data set had a likelihood ratio p-value of 0.060, while the others were all ≥0.20. The estimated odds ratios per 10 year increase in age were 1.17 for the stage 0 to 1 transition, 1.81 for the 1 to 2 transition, 0.98 for the 2 to 3 transition, and 1.75 for the 3 for 4 transition.

**Table 7:** Estimated effects of age when added to the primary model from Table 4, for the original data and for simulated data with no age effects.

		Odds	95% confidence interval		
Predictor(s)^a^	Value	Ratio	Lower	Upper	P-value
Models of the original, actual data					
Current age at each time step	Per 10 years	1.33	1.15	1.54	0.0002
Age at HCV infection	Per 10 years	1.31	1.13	1.52	0.0003
Current age at each time step	Per 10 years	1.45	0.74	2.8	0.28
Age at HCV infection	Per 10 years	0.92	0.47	1.79	0.80
Models of simulated data with no actual age effects					
Current age at each time step	Per 10 years	1.08	0.88	1.31	0.46
Age at HCV infection	Per 10 years	1.07	0.90	1.27	0.45
Replication on another independently simulated set of data with no actual age effects					
Current age at each time step	Per 10 years	1.03	0.87	1.22	0.74
Age at HCV infection	Per 10 years	1.02	0.87	1.20	0.77

a Results separated by vertical space are from separate models; one model included both current age and age at HCV infection.

Abbreviations: HCV: hepatitis C virus.

Because error in imputed ages at HCV infection could bias estimated age effects as described in the Methods, we evaluated the potential magnitude of such bias by analyzing simulated data sets that had realistic amounts of error in age at HCV infection and were generated from models with no actual age effects. Two replicates of the process, shown in Table 7, had only small estimated age effects, suggesting that most of the estimated effects for the actual data are unlikely to be due to bias.

## Discussion

We analyzed a substantial amount of data on fibrosis progression using a new method that avoids many problems inherent in other methods that have been used to analyze such data. We found evidence that progression risk decreases after more time has been spent in a stage, which concords with an analysis of progression following liver transplant that used the same methods [10]; methods previously used for analyzing fibrosis progression cannot assess such effects. This finding may reflect a dynamic nature of HCV infection, with recent progression indicating active disease and a higher risk of further progression. Older age increased progression, and this appeared to be driven by current age rather than being a fixed effect of age at HCV infection (the evidence for this is not conclusive, however, as shown by the wide confidence intervals in the model in Table 7 that includes both age effects). This also accorded with the previous analysis of post-transplant progression, where progression increased with donor age. Other previous analyses have also found an age effect [25] but were limited by their methodology to evaluating presumed age at HCV infection rather than current age; they also did not recognize or assess potential bias [5]. A small simulation experiment indicated that little of our observed age effect appeared to be due to bias. We also found evidence for a protective effect of African American race, which has previously been suspected [9]. There was a suggestion of increased risk due to HIV co-infection, particularly in the era of effective anti-retroviral therapy beginning in 1996. In prior years, HIV-infected potential participants who experienced accelerated HCV progression may have also had higher mortality from opportunistic infections, causing them to be excluded from our source studies. There was also a suggestion that slower progression through stage 0 tends to be followed by slower progression through stage 1, but this did not hold for later transitions between stages. A number of other factors may influence progression, as some estimates in Table 5 may be large enough to be important (e.g., male sex, heavy alcohol consumption, and body mass index >30) and upper confidence bounds generally are not low enough to provide strong evidence against substantial increased risk.

The results here may seem to be less reliable than previous studies of similar data because of the complex methods and assumptions. The complexity, however, is inherent in the available data and the disease process. Previous studies only appear to avoid this by making strong simplifying assumptions that are implicit or not given strong emphasis. Consider, for example, the simple approach of obtaining a single fibrosis rate per year for each person by dividing current observed stage by the time since presumed infection [25]. This implicitly assumes that infection is immediate at the reported time of first risk with no inaccuracy in those reported times, fibrosis is never misclassified, each progression between stages is numerically equivalent, and the observed stage was just reached at the time of biopsy. Each of these assumptions simplifies the statistical analysis and reduces the apparent statistical variation in resulting estimates, but each is also questionable or even known to be wrong. We have attempted to deal realistically with these complexities. Notably, using multiple imputation [11] to address the unknown ages at HCV infection adds considerable complexity and results in wider confidence intervals than would have been produced by pretending that ages of HCV infection were all known, but this uncertainty really does exist. We have also used multistate models, which better match biopsy-based measurement of the disease process, and have evaluated departures from the Markov assumptions usually used in multistate modeling. We checked linearity and variation by stage for key predictors, and sensitivity analyses suggested that our main results did not rely on the particular size of time step, misclassification probabilities, or imputation model for age of HCV infection. We believe that all these facts add credence to our results.

Our methods permit analysis of time-varying covariates, which was important for HIV and age. The distinction between current age (time-varying) and age at HCV infection (fixed) may seem subtle, but they could have different implications for the biology of HCV disease and also for clinical prognosis. For example, detecting recent progression to higher fibrosis could be cause for alarm in an older patient even if original HCV infection was at a very young age. This is the second infectious disease for which one of us (PB) has found that careful consideration of both fixed and time-varying age effects points to a different conclusion than only considering fixed effects [26].

### Limitations

Despite the specialized analyses and other strengths, this study has a number of limitations. As for many other studies using liver biopsies, selection bias is a potential concern. Restricting study to clinic populations or those already known to be HCV infected can create selection bias toward more rapid progression [27], and the SFVA and HALS groups are clinic-based or partly clinic-based. For ALIVE, there should be little selection bias, because participants were selected from the community without respect to HCV status, enrolled participants were tested for HCV, and a random sample of those found to be chronically infected underwent biopsy [14]. For all three studies, participants had to agree to undergo liver biopsy in order to be included; this could select for more severe disease if participants were more likely to agree if they had symptoms. On the other hand, the unavoidable restriction to participants who were alive at the time of recruitment could tend to exclude more rapid progressors. Statistical methods for dealing with this, known as *left-truncation* or *late entry*, are available but would require a model that includes death from fibrosis progression as an additional stage; calculations to deal with late entry are also not available in the mspath software. Our selection of only followup before treatment with interferon could also tend to exclude more rapid progressors, although this may be mitigated by the usual clinical desire to obtain a biopsy before starting treatment. Selection bias may be most important for estimation of overall rapidity of progression rates, which we have not emphasized. For the estimated effects of risk factors to be biased, selection for greater severity would have to differ according to the levels of the risk factors. Because selection likely did differ between source studies, we took steps to fully control for the effect of study (see next paragraph).

The distribution of biopsy-measured fibrosis was heterogeneous across source studies. The strong influence of source study, and its variation by stage, likely result from non-biological influences. Differing misclassification of fibrosis due to differing readings of biopsies is one important possibility, but differing selection of participants is also likely to contribute to the study effects. For example, it is unlikely that the true rate of stage 4 (cirrhosis) was really the same in HALS as in the other studies; this would imply that about 10 biopsies showing cirrhosis were all misread in HALS. Because the studies were drawn from differing populations by differing methods, selection effects are bound to differ between them. The proportions of biopsies from different calendar time periods also differed between studies. The imputation models for age at HCV infection accounted for strong influences of calendar time [5]; changes over time in progression rates may also be possible, but seem less likely with our focus on pre-treatment biopsies only. We minimized the potential for source study to confound other associations by fully controlling for its influence on every transition, using a full contingent of 8 parameters for study effects. This reduced the potential influence of selection bias on other estimates, but it also added complexity to the models and may have reduced the precision of other estimates. Using fewer parameters did not seem viable, because differences between the studies varied by stage, and because any oversimplification of study effects could increase concern about selection bias and confounding.

We accounted for possible misclassification of biopsy-measured fibrosis by factoring external estimates of misclassification rates into the estimation process. This increases the statistical uncertainty in our results, but rightly so. Along with multiple rather than single imputation of age at HCV infection, this results in a better assessment of precision and is a strength of this analysis. In addition, a sensitivity analysis showed that our main findings were insensitive to the exact misclassification assumptions. Nevertheless, an ideal approach would utilize information on biopsy quality to provide a customized matrix of misclassification probabilities for each biopsy. Unfortunately, we did not have such refined estimates available. Some multistate modeling methods, including the one used here, can estimate misclassification probabilities as part of the modeling process. This, however, would be computationally very challenging and seems likely to be less accurate than estimates from studies that were focused specifically on misclassification and therefore employed multiple readings of the same biopsy or multiple biopsies of the same liver. We have therefore used the best estimates from such studies that we could obtain [4]. The median (interquartile range) biopsy length was 19mm (14–24) in HALS and 12 mm (9–19) in ALIVE, which are comparable to those in the studies used to estimate misclassification [4]. (Biopsy length was not available for the SFVA study.)

We assumed no backward transitions to lower stages; our restriction to pre-treatment biopsies may make this assumption reasonable, and no participants had spontaneously cleared HCV infection at the time of any biopsy. This assumption implies that any apparent backward transitions must be ascribed to misclassification of at least one of the biopsies. Among the 159 participants with two or three biopsies, 21% had nominal backward transitions of one stage and 3% of two stages, rates that seem to be readily explainable by the substantial misclassification probabilities previously estimated [4]. The progression model could in principle be extended to allow backward transitions, and the mspath software allows any specification of what transitions are possible. Unfortunately, allowing backward transitions would vastly increase the number of possible paths to be evaluated, making computations infeasible. In addition, parameters governing the four additional transitions would have to be added, and factors influencing regression could differ from those for progression, substantially complicating the modeling. We therefore cannot evaluate the effect of allowing backward transitions on our results, but we believe that excluding them is close enough to reality that it is unlikely to introduce serious bias.

A reviewer pointed out a potential bias that could impact our estimated effects of years in prior stages, at the bottom of Table 4. For example, if a participant were known to have reached stage 2 by a given time, with an unknown time of transition from stage 0 to 1, then a shorter time in stage 0 would mean a longer time in stage 1 before progressing to stage 2, while a longer time in stage zero would mean a shorter time in stage 1 before progressing to stage 2. Either way would contribute to an apparent effect of longer time in stage 0 increasing risk of progression from stage 1 to 2. Fortunately, we observed the opposite of what this bias would produce, instead estimating a *protective* effect of longer time in stage zero. Thus, the possibility of this bias only strengthens the evidence for this effect. For the 2 to 3 and 3 to 4 transitions, we did estimate increases in risk such as this bias would produce. This bias is directly analogous to the potential bias in the estimated effect of age at infection that we described, with time in previous stages playing the role of time before (i.e., age at) HCV infection, so a similar simulation-based investigation could be undertaken. We do not consider this to be worthwhile, however, because the issue is largely overshadowed by the very wide confidence intervals for these effects.

Most of our participants had only one biopsy, which made them less informative for estimation of the effects of years in current stage and years in previous stages. Fortunately, we had enough with multiple biopsies (15%) to obtain some useful estimates, notably the protective effects of years in current stage in Table 4 for the 0 to 1 and 2 to 3 transitions. We also had relatively few participants with advanced fibrosis, which is reflected in extreme estimates or wide confidence intervals for most estimated effects that are specific to the 2 to 3 transition and the 3 to 4 transition. Some predictors may have been inaccurately measured, due to reliance on self-report and extrapolation of study values to the entire period of HCV infection, and some were missing for a considerable proportion of participants. We did not analyze HCV genotype or viral load as predictors, and complete history of alcohol consumption was only available in the HALS study. We did not evaluate the influence of measured immune status and antiretroviral treatment history directly for HIV-infected participants. This would be very complicated and could be distorted by self-selection of treatment and incomplete histories; we investigated differing HIV effects over calendar time as a feasible alternative. Our statistical methods model progression over the entire period of HCV infection, so we could not evaluate factors such as inflammation and steatosis grade that are known only at the time biopsy; these would have to be known at all times and treated as time-varying covariates, but are unlikely to have remained constant since infection. Our assumptions concerning fibrosis misclassification and our model for imputing age of HCV infection could be inaccurate, but sensitivity analyses using alternatives showed similar results. All of our fitted models had some parameters estimated to be effectively infinite. These could not be used in standard methods for multiple imputation analysis, so we used only the finite estimates and reported the infinite estimates separately. In some cases, standard errors for other parameters had to be obtained by re-estimating models with the effectively infinite parameters held fixed; those standard errors did not appear to differ substantially from the cases where standard errors were available despite estimation of some effectively infinite parameters. Finally, the computational burden of the method we used is substantial. We were only able to complete our analyses within about a month by often running 20 or more analyses simultaneously using a specialized parallel computing facility.

### Further Research

Ideally, prospective followup of persons known to be recently infected with HCV would prevent selection biases and maximize the value of information obtained from biopsies. Performing such studies, however, might be difficult and expensive. Steps to minimize misclassification (e.g., using multiple readings of each biopsy) could also make data more informative and potentially reduce the computational burden of our methods (if some misclassifications become impossible). The methods used here might provide more credible results and additional insights if applied to larger data sets with more repeat biopsies. Computational feasibility is a potential issue, but will improve with time. The non-Markov multistate modeling software that we used is freely available at http://cran.r-project.org/web/packages/mspath/index.html.

An appealing alternative to biopsy is non-invasive measurement of fibrosis via imaging or biochemical analysis of peripheral blood samples [28], [29]. Some methods may already be as accurate as biopsy, but evaluation of alternatives has suffered from inappropriate use of biopsy as a gold standard [30]. Studies with frequent non-invasive measurements could be less dependent on imperfectly known times of HCV infection, because they could better focus on observed trajectories. Evaluating how recent changes in non-invasive measures predict subsequent change would permit exploration of the phenomenon we found of decreasing progression risk with longer times already spent in a stage. If the invasiveness, risk [31], and expense of biopsy curtails its use in HCV research, the methods used here may still be useful for analysis of data for other diseases that progress through stages.
